# *DRD4* Polymorphism Moderates the Effect of Alcohol Consumption on Social Bonding

**DOI:** 10.1371/journal.pone.0028914

**Published:** 2012-02-08

**Authors:** Kasey G. Creswell, Michael A. Sayette, Stephen B. Manuck, Robert E. Ferrell, Shirley Y. Hill, John D. Dimoff

**Affiliations:** 1 Department of Psychology, University of Pittsburgh, Pittsburgh, Pennsylvania, United States of America; 2 Department of Human Genetics, University of Pittsburgh, Pittsburgh, Pennsylvania, United States of America; 3 Department of Psychiatry, University of Pittsburgh, Pittsburgh, Pennsylvania, United States of America; University of Granada, Spain

## Abstract

Development of interpersonal relationships is a fundamental human motivation, and behaviors facilitating social bonding are prized. Some individuals experience enhanced reward from alcohol in social contexts and may be at heightened risk for developing and maintaining problematic drinking. We employed a 3 (group beverage condition) ×2 (genotype) design (N = 422) to test the moderating influence of the dopamine D4 receptor gene (*DRD4* VNTR) polymorphism on the effects of alcohol on social bonding. A significant gene x environment interaction showed that carriers of at least one copy of the 7-repeat allele reported higher social bonding in the alcohol, relative to placebo or control conditions, whereas alcohol did not affect ratings of 7-absent allele carriers. Carriers of the 7-repeat allele were especially sensitive to alcohol's effects on social bonding. These data converge with other recent gene-environment interaction findings implicating the *DRD4* polymorphism in the development of alcohol use disorders, and results suggest a specific pathway by which social factors may increase risk for problematic drinking among 7-repeat carriers. More generally, our findings highlight the potential utility of employing transdisciplinary methods that integrate genetic methodologies, social psychology, and addiction theory to improve theories of alcohol use and abuse.

## Introduction

Social factors play an instrumental role in the development and maintenance of alcohol use disorders [1], [2]. Older adolescents and young adults do nearly all of their drinking with others [3], [4], suggesting that social processes may be particularly important in shaping drinking behavior early on and may play a key role in the development of problematic drinking [5]. Surveys indicate that people commonly endorse social motives for drinking [6]–[8], and expectancies of social facilitation are especially powerful in young adult drinkers [9], [10]. Moreover, the belief that alcohol facilitates social functioning is associated with problematic drinking in cross-sectional studies [11]–[13] and, in prospective studies, predictive of actual alcohol use [14] and alcohol use disorders. For instance, Patrick and colleagues [15] showed that social/recreational reasons for drinking at age 18 predicted symptoms of alcohol use disorders 17 years later, and Beseler and colleagues [16] showed that adults with a family history of alcoholism who drank for social facilitation and to reduce negative affect had a greater risk of alcohol dependence 10 years later.

Despite the general importance of social factors in the etiology of alcohol use disorders, there likely are individual differences in the extent to which alcohol is socially reinforcing. Individuals who experience more reward from alcohol in social settings may be at increased risk to misuse alcohol [17], [18], suggesting that individual differences in the socially reinforcing effects of alcohol may be related to genetic makeup. Social contexts can moderate the impact of genetic risk factors for a wide range of psychopathologies [19] including alcohol-related traits [20]. Indeed, the “contextual triggering” model of Shanahan & Hofer [21] states that social contexts can trigger a genetic predisposition. The social context in which drinking occurs may be an especially salient environmental factor with potential to modulate genetic influences on alcohol response [22]–[24]. Surprisingly, experimental paradigms designed to examine the reinforcing effects of alcohol have largely failed to consider social context. These laboratory studies recruit participants who almost always drink in social settings [3], but nearly all test these social drinkers in isolation [17]. Accordingly, most studies create uncommon conditions to assess the reinforcing effects of alcohol. Without considering social context, it is unsurprising that investigators have struggled to reliably explain the reinforcing effects of alcohol [25] or genetic mechanisms underlying these effects [26].

Group settings offer a unique chance to uncover important reinforcing effects of alcohol that might otherwise go unnoticed when examining participants in isolation [17], [27]. In fact, many of the subjectively pleasant effects of alcohol that confer increased risk for alcohol misuse (e.g., increased sociability) must be studied in a group setting [28]. There has been little systematic research on the effects of alcohol conducted in group settings, though, and despite the noted importance of contextual variables in the study of genetic effects [29], [30], no prior laboratory study has examined the moderating role of genetic variation on alcohol's reinforcing effects in a controlled group setting.

Because both the reinforcing effects of alcohol [31] and the rewarding effects of social interactions [32] are mediated via dopamine-dependent activity of the brain's mesocorticolimbic reward system, polymorphic variations in dopamine-regulating genes offer rational candidates for the genetic study of problematic drinking [33] and the study of interactions between alcohol abuse and social behaviors [34]. One particularly prominent polymorphism in psychiatric and behavioral genetics consists of a Variable Number of Tandem Repeats (VNTR) in exon 3 of the gene encoding the dopamine D4 receptor (*DRD4*), represented by common length variants of 2, 4, and 7 repeats in most populations [35]. Activation of the G-protein-linked D4 receptor attenuates intracellular signaling by inhibiting adenylyl cyclase coupling, and this inhibitory effect is blunted by presence of the 7-repeat allele [36]–[38]. It is this attenuated response to dopamine produced by the 7-repeat variant that putatively underlies hypothesized associations of this polymorphism with addiction-related phenotypes [39], [40].

The 7-repeat allele of the *DRD4* polymorphism has been associated with several behaviors and experiences, such as cigarette smoking [41]–[43], cue-elicited craving [44]–[47] but see [48], pathological gambling [49], [50], laboratory measures of financial risk taking and inhibitory motor control (e.g., [51]–[54]), fairness preference [55], human assortative mating patterns [56], and infidelity/sexual promiscuity [57], as well as disorders, such as Attention Deficit/Hyperactivity Disorder (ADHD) [58]–[60]. Notably, too, a growing literature shows many developmental effects of this VNTR on early behavioral outcomes (e.g., attachment organization, externalizing disorders, sensation seeking, and prosocial behaviors) to vary as a function of naturally occurring or experimentally manipulated environmental exposures [61], which in turn marks this polymorphism as a prime candidate for gene-environment interaction. In particular, the *DRD4* genotype pertains to gene-environment interactions involving alcohol-related traits [62], [63].

Two recent studies underscore the importance of social factors in the link between *DRD4* genotype and alcohol outcomes. Larsen et al. [62] reported that individuals carrying the 7-repeat allele drank more in the presence of a heavy-drinking confederate than those of other *DRD4* genotypes, and Park et al. [63] found college/Greek involvement to be associated with increased risk of alcohol dependence, but only among students with at least one copy of the 7-repeat allele. Taken together, these two studies conducted in two different laboratories suggest a gene- environment interaction, such that the *DRD4* VNTR is associated with problematic drinking only in the presence of certain social-environmental factors (specifically, heavy drinking peers and college/Greek involvement). The pathways by which social factors increase risk for problematic drinking among 7-repeat carriers have yet to be articulated. As noted by Park et al. [63], “Specific factors in college environments that interact with the *DRD4* gene to increase alcohol dependence in emerging adulthood need to be identified.”

One factor of particular relevance to young adults is the formation of social bonds [64]. To our knowledge, however, no prior study has examined whether effects of alcohol on social bonding may be moderated by *DRD4* variation (or any other gene polymorphism). Accordingly, we sought to extend the findings of Larsen et al. [62] and Park et al. [63] to investigate whether experimentally manipulated alcohol consumption would promote social bonding in randomly assigned groups of three unacquainted young adults and would do so differentially among those of differing *DRD4* genotype. Each three-person group was assigned to one of three beverage conditions (alcohol, placebo, or non-alcohol control) (i.e., all participants in each group were assigned to the same beverage condition). Within each condition, participants were grouped by presence or absence of the *DRD4* 7-repeat allele. We hypothesized that alcohol would increase perceived social bonding and that individuals carrying the 7-repeat allele would be especially sensitive to alcohol's effects on social bonding.

## Methods

### Ethics Statement

Each participant gave informed written consent to take part in this study. All aspects of this research were approved by University of Pittsburgh's Institutional Review Board.

### Participants and design

Male and female social drinkers (n =  720) aged 21–28 were recruited via community and university newspaper ads for a parent study of the effects of alcohol on social bonding [65]. A subset of Caucasian participants (*n* = 422) were genotyped for the *DRD4* VNTR. Exclusion criteria included a history of adverse reaction to the type or amount of beverage used in the study, any medical conditions that contraindicated alcohol administration, meeting criteria for past alcohol abuse or dependence, as indexed by the *Diagnostic and Statistical Manual of Mental Disorders* [66], a weight not within 15% of ideal weight for their height [67], illiteracy, pregnancy in females, and smoking 15 or more cigarettes/day (to avoid nicotine withdrawal). Inclusion criteria included drinking a mean of at least two drinks on at least one occasion per 2 weeks, or at least four drinks on at least one occasion per month, over the past year. Participants who consumed alcohol could not drive themselves home from the study.

Study sessions took place on a weekday (Monday-Friday), with the group drinking period beginning at approximately 12 PM. Participants were randomly assigned to groups of three unacquainted persons, and these groups were randomly assigned to drink over 36-min a moderate dose of alcohol, a placebo, or a non-alcoholic control drink (additional details provided below). After drinking, participants were separated, and each completed the Perceived Group Reinforcement Scale (PGRS; described below) and several other measures unrelated to social bonding, which are not reported here.

### Genotyping and procedure

Saliva was collected using Oragene kits (DNA Genotek, Ottawa), and genomic DNA was isolated following the manufacturer's protocol. The 48 bp VNTR in Exon 3 of *DRD4* was genotyped by the method of Lichter et al. [68], and genotypes were assigned by direct comparison to controls of known genotype. Allele and genotype frequencies are presented in Table 1. Allele frequencies were in Hardy-Weinberg Equilibrium (*p* = .56). Due to the low frequency of individuals homozygous for the 7-repeat allele (2.6%) and in accordance with prior convention (e.g., [62]), participants were classified as 7-present (i.e., homozygous or heterozygous for the 7-repeat allele) or 7-absent (i.e., neither allele is 7-repeat). Most studies examining an association between the *DRD4* VNTR and a multitude of disorders and traits, including alcohol-related phenotypes, have assumed that a linear association exists between repeat length and functionality. We rely, however, on data indicating that this is unlikely, with 10 repeats functionally resembling 2 repeats more so than 7 repeats [36]–[37], [69]. Regardless, there were only 13 individuals (3%) with >7 repeats in our sample, and results were unchanged when using the long/short classification of alleles (i.e., including individuals with repeats >7 in the 7-present classification presented here). As depicted in Table 2, *DRD4* genotypes were evenly distributed across beverage conditions, χ^2^(*df = *2, *N* = 422) = 3.25, *p* = .20.

**Table 1:** DRD4 VNTR Allele and Genotype Frequencies.

Allele/Genotype	*n*	%
Allele		
2	70	8.30
3	31	3.67
4	547	64.81
5	11	1.30
7	172	20.38
8	13	1.54
Total	844	100
Genotype		
2/2	2	0.47
2/3	1	0.24
2/4	47	11.14
2/7	17	4.03
2/8	1	0.24
3/3	1	0.24
3/4	17	4.03
3/7	10	2.37
3/8	1	0.24
4/4	175	41.47
4/5	7	1.66
4/7	117	27.73
4/8	9	2.13
5/7	4	0.94
7/7	11	2.60
7/8	2	0.47
Total	422	100
Genotype Classification		
7-present	161	38.15
7-absent	261	61.85
Total	422	100

**Table 2:** DRD4 Genotype Distribution Across Beverage Conditions.

	Alcohol		Placebo		Control		Total	
	*n*	*%*	*n*	*%*	*n*	*%*	*n*	*%*
7-present	68	43.31	42	33.07	51	36.96	161	38.15
7-absent	89	56.69	85	66.93	87	63.04	261	61.85
Total	157	100	127	100	138	100	422	100

#### Predrink assessment

Before group formation, participants completed the NEO Five-factor Inventory (NEO-FFI) [70], which reliably assesses five domains of adult personality (neuroticism, extraversion, openness to experience, agreeableness, and conscientiousness), and the Biphasic Alcohol Effects Scale (BAES) [71], which includes seven items that assess feelings of stimulation (e.g., energized, excited), and seven that assess feelings of sedation (e.g., heavy head, difficulty concentrating). Several steps were taken to ensure that the groups included 3 unacquainted participants (using methods previously employed in our lab) [17]. An initial blood alcohol content (BAC) breath sample was obtained, and participants completed a subjective intoxication scale (SIS) on which 0 meant *not at all intoxicated* and 100 meant *the most intoxicated I have ever been*.

#### Drink administration

Group members were informed that they would consume their drinks together before they would complete tasks related to memory and cognitive performance (the ostensible study aim). Participants were told that the group drinking format made it easier to monitor their beverage consumption. All participants in each group drank their beverages seated around a circular table (see [17]). Participants were asked not to mention how intoxicated they might be feeling. To increase credibility in the placebo condition, drinks were mixed in front of participants and the glass was smeared with vodka [72]. The alcoholic beverage was 1 part vodka and 3.5 parts cranberry juice cocktail (Ocean Spray). For those drinking alcohol, the vodka bottle contained 100-proof vodka (Smirnoff); for those drinking a placebo, the vodka bottle contained flattened tonic water (Schweppes). Control participants were told they did not receive alcohol and were given cranberry juice in equal volume. After participants were given one third of the drink [alcohol participants were given one third of a moderate dose of alcohol (0.82 g/kg males/0.74 g/kg females)] and asked to consume it evenly over 12 min, the experimenter exited the room. The experimenter re-entered the room just before the end of each 12-min drinking block (at 12- and 24-min) to give participants the middle and final thirds of the drink. During each pour, participants were asked to consume the beverage evenly over 12-min intervals. Other than briefly entering the room to fill participants' glasses, the experimenter was not present during the group drink period.

#### Postdrink assessment

After drinking the final third (36-min), participants were separated and BAC and SIS ratings were recorded. To help control for dosage set, placebo participants received a BAC reading ranging from .041% to .043% (randomly assigned), which is about the highest credible reading for deceived participants (see [72]). This false reading aids in placebo deception [73] (actual BAC readings were also recorded). Participants then completed the Perceived Group Reinforcement Scale (PGRS) to assess the perception of social bonding and the BAES. The PGRS included 12 items, such as “*I like this group”* and *“The members of this group are interested in what I have to say,*” which were summarized as a composite score (Cronbach's α = .90). Items were adapted from the Group Attitude Scale [74] and the Perceived Cohesion Scale [75]. The PGRS has good face validity (see Table 3 for the individual items comprising the scale), and it has proven sensitive to the effects of alcohol on social bonding in our prior research. Importantly, the PGRS demonstrates good convergent validity as well, as it correlates with other non-verbal measures of social bonding (see [17]). BAC and SIS were again obtained about 10-min after completing these scales. Placebo participants were presented with a false BAC reading between .039% and .037% and, along with control participants, were debriefed, paid $60, and allowed to leave. Alcohol participants recorded their BACs and ate lunch/relaxed. When their BACs dropped below .025%, they were debriefed, paid $60, and allowed to leave (they were not permitted to drive).

**Table 3:** The Perceived Group Reinforcement Scale (PGRS).

1.	I like this group.
2.	The members of this group are interested in what I have to say.
3.	The members of this group value my ability to contribute.
4.	My presence makes a difference to this group.
5.	I see myself as an important part of this group.
6.	I am satisfied with this group.
7.	The members of this group underestimate my ability to contribute.
8.	I often disagree with the members of this group.
9.	I feel included in this group.
10.	In spite of individual differences, a feeling of unity exists in this group.
11.	My presence is irrelevant to this group.
12.	If an opportunity occurred outside this lab, I would look forward to being part of this group in the future.

Note: Each item was rated on a scale ranging from 1 (“strongly agree”) to 9 (“strongly disagree”). Item numbers 7, 8, and 11 were reverse scored.

## Results

Participants (males = 51.4%) had a mean age of 22.4 years (SD = 1.8). Gender, age, marital status, income, felt stimulation/sedation prior to drinking (as assessed by the BAES), prior drinking patterns, extraversion (along with the other 4 personality dimensions on the NEO-FFI), and smoking status were equivalent across drink conditions. Participants drank on average slightly more than twice a week [M =  3.78 (SD = 0.90) using a 7-point scale with “3” = 1–2 occasions/week and “4” = 2–3 occasions/week] and consumed an average of 4.32 (*SD* = 1.92) drinks per occasion.

BACs and SIS scores recorded after drinking and after completing the PGRS and BAES across drink conditions appear in Table 4. Mean BAC values indicate alcohol participants were on the ascending limb of the BAC curve with a BAC about .06% when completing the PGRS and BAES. As expected, placebo participants felt significantly more intoxicated than control participants and significantly less intoxicated than alcohol participants.

**Table 4:** Beverage Response Variables.

	Alcohol		Placebo		Control		
Characteristic	*Mean*	*SD*	*Mean*	*SD*	*Mean*	*SD*	*F*
BAC post-drink	0.054*^a^*	0.012	0.001*^b^*	0.001	0.001*^b^*	0.001	2649.51**
BAC post-PGRS†	0.062*^a^*	0.011	0.001*^b^*	0.001	-----	-----	3896.09**
SIS post-drink	38.39*^a^*	16.89	15.26*^b^*	10.31	0.09*^c^*	0.73	396.44**
SIS post-PGRS†	34.75*^a^*	16.53	9.85*^b^*	11.34	-----	-----	208.63**

*p = <.05.

**p = <.001

†analyses did not include control participants as they were not asked to provide these data

*Note.* PGRS =  Perceived Group Reinforcement Scale. BAC = blood alcohol concentration. SIS = subjective intoxication scale (values ranging from 0 to 100).

### 

#### Statistical Analysis

Given the hierarchical structure of the data (each individual is nested within one drinking group), it is important to account for the potential interdependence of participants' PGRS responses [76]. The intraclass correlation coefficient (ICC), which assesses the degree of clustering or non-independence of PGRS scores among group members, was calculated to be .23. This ICC value indicates that there is substantial clustering of PGRS scores within groups [77], which violates a key assumption of the statistical model used by ANOVA (i.e., independence of observations). As such, a hierarchical linear model was used to model PGRS scores (a continuous variable) by beverage content condition (three levels: alcohol, placebo, or control) and *DRD4* (two levels: 7-present vs. 7-absent) using the SAS PROC MIXED procedure. Because group size was small (n = 3), intercepts but not slopes were allowed to vary randomly across groups [76]. As noted by Kenny and colleagues [78], it is the variation in these intercepts that models the non-independence of groups (pg. 132).

### Drink Condition and *DRD4* Genotype Effects on Social Bonding

**Figure 1:**
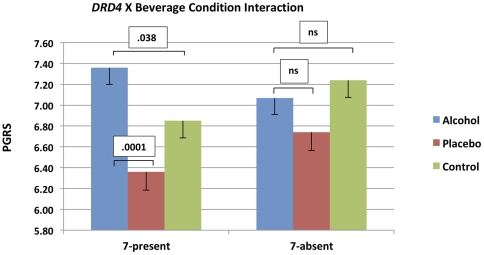
PGRS Scores (Mean, SE) by DRD4 Genotype and Beverage Condition.

The tests of fixed effects are depicted in Table 5. A main effect of beverage condition on PGRS ratings (*p* = .001) revealed that alcohol participants reported higher PGRS scores than placebo participants (*p* = . 0003), but similar scores to control participants (*p* = .36). In addition, control participants reported higher PGRS scores than placebo participants (*p* = .008). As expected, there was no main effect of *DRD4* on PGRS scores (p = .24). Importantly, there was a significant 3 (BEVERAGE)×2 (*DRD4*) interaction (*p* = .022). PGRS scores (*M*±*SE*) across beverage conditions and genotypes are shown in Table 6. As depicted in Figure 1, 7-repeat carriers reported higher PGRS ratings in the alcohol condition than in either the placebo (*p*<.0001) or control conditions (*p*<.04), whereas alcohol did not significantly affect ratings of 7-absent carriers. The current model explained 3% of level-1 variance and 13% of level-2 variance (represented by proportional reductions in the variance-component residual in comparison with the empty model without explanatory variables; [79]). Table 7 shows the variance components and goodness of fit characteristics associated with each model we estimated. As can be seen, the model with the interaction term included provided a significantly better fit to the data than both the empty model and the model including only main effect estimates.

**Table 5:** Tests of Fixed Effects: Results of Hierarchical Linear Modeling.

Effect	Numerator *df*	Denominator *df*	F value	*p* value
Beverage	2	215	7.11	0.001
*DRD4*	1	395	1.41	0.237
*DRD4*×Beverage	2	394	3.86	0.022

**Table 6:** PGRS scores (M±SE) by Beverage Condition and Genotype.

	Alcohol	Placebo	Control	*Genotype Mean*
7-present	7.37 (.17)	6.35 (.20)	6.86 (.18)	6.86 (.10)
7-absent	7.04 (.15)	6.75 (.15)	7.24 (.15)	7.01 (.09)
*Beverage Mean*	7.21 (.11)	6.55 (.13)	7.05 (.13)	

*Note.* Possible range = 1–9. Contrasts examining carriers and non-carriers within each beverage condition failed to reach significance.

**Table 7:** Variance Components and Model Fit.

	Empty Model *Coef (SE)*	*DRD4 Coef (SE)*	*DRD4* and Drink *Coef (SE)*	*DRD4*×Drink *Coef (SE)*
***Variance Components***				
Variance in group intercepts	0.46(.68)	0.46(.68)	0.39(.63)	0.40(.63)
Variance within groups	1.30(1.14)	1.30(1.14)	1.30(1.14)	1.26(1.13)
***Goodness of Fit***				
No. of Parameters	3	4	6	8
Deviance (FIML)	1419.93	1419.38	1407.46	1399.82
Chi-square statistic		0.55	11.93	7.64
Degrees of freedom		1	2	2
P-value		>0.50	0.003	0.021

As a supplementary analysis, we collapsed across the two nonalcohol conditions (the placebo and control groups) and modeled PGRS scores by beverage content condition (two levels: alcohol vs. no-alcohol) and *DRD4* (two levels: 7-present vs. 7-absent) using the SAS PROC MIXED procedure. Alcohol participants reported higher PGRS scores (M = 7.2, SE = .12) than no-alcohol participants (M = 6.8, SE = .09), (*F* (1, 203) = 6.75, *p* = .01). Again, there was no main effect of *DRD4* on PGRS scores (p = .8). There was a significant 2 (BEVERAGE)×2 (*DRD4*) interaction, *F* (1, 405) = 7.42, *p* = .007). Consistent with the results above, 7-present individuals reported higher PGRS ratings in the alcohol-consuming condition (M = 7.4, SE = .17) than in the no-alcohol consuming condition (M = 6.6, SE = .14; p = .0006), whereas alcohol consumption did not significantly affect ratings of 7-absent carriers (alcohol; M = 7.0, SE = .15: no-alcohol; M = 7.0, SE = .11; p = .82).

Gender, age, extraversion (along with the other 4 personality dimensions on the NEO-FFI), and drinking history were equivalent across genotypes. Additionally, among those drinking alcohol, there were no differences between 7-present and 7-absent genotypes on BACs, on ratings of subjective intoxication (as assessed by the SIS), and on felt stimulation and sedation (as assessed by the BAES) after alcohol consumption. These results suggest that the findings are unlikely due to systematic differences between the two genotype groups on the above mentioned variables.

## Discussion

This study provides initial evidence for a moderating effect of the *DRD4* polymorphism on the relationship between alcohol consumption and social bonding. 7-present individuals reported increased perceived social bonding in an unstructured group setting after drinking alcohol, compared to placebo and non-alcohol control beverages. In contrast, alcohol did not affect perceived social bonding of 7-absent individuals. Our findings converge with and extend those of Larsen et al. [62] and of Park et al. [63] suggesting that *DRD4* may be linked to the development of problematic drinking partly through the formation of social relationships. Developing interpersonal relationships is a fundamental human motivation [64], and behaviors that support the formation of social bonds are highly rewarding (e.g., [80]). Our results suggest that one possible pathway by which alcohol may become more reinforcing for 7-repeat carriers is by the facilitation of perceived social bonding.

The current study is the first to examine the impact of genetic variation, alcohol consumption, and perceived social bonding among previously unacquainted individuals in a controlled group setting. Because the typical effect size for genetic variation acting on behavioral phenotypes is small [21], large samples are necessary to draw firm conclusions about how certain polymorphisms modulate the experience of alcohol. In one of the largest alcohol administration studies yet conducted, we were able to detect small though potentially meaningful genetic effects. Use of an ecologically valid social drinking context, in which unacquainted young adults consumed alcohol together, increases the generalizability of our results to the natural environment. More generally, this design, which manipulated the environment through random assignment, uniquely allows us to draw causal inferences regarding this gene- environment interaction [81].

Social reward and the reinforcing effects of drugs of abuse, including alcohol, are mediated in part through the mesocorticolimbic dopamine system [32], and recent studies have focused on the role of dopamine in regulating interactions between alcohol and social factors (e.g., [34]). Because 7-repeat carriers may be more sensitive to the dopamine response triggered by priming doses of alcohol and alcohol-related cues [45], [47], [62], they may perceive enhanced social bonding while drinking due to an augmented dopamine response in the brain's reward circuitry. This explanation is generally consistent with prior reports showing that 7-present individuals respond to alcohol consumption with increased craving (e.g., [45]) and respond to positive-feedback with increased reward-related reactivity in the ventral striatum [82] compared to 7-absent individuals.

Consistent with prior studies [45], [47], 7-present individuals did not report feeling more intoxicated nor did they report more stimulation (e.g., elated, energetic, excited) after alcohol consumption, indicating that 7-present individuals did not appear to be generally feeling more of the euphoric effects of alcohol on the ascending limb of absorption than 7-absent individuals. Rather, our results suggest a separate pathway by which alcohol becomes more rewarding for 7-present individuals by increasing their perceived ability to bond with their peers. Future work should examine the relationship between increased stimulation/euphoria and enhanced perception of social bonding more fully, though, as these results are based only on the BAES. Furthermore, we found a statistical trend for a difference in perceived social bonding between 7-repeat carriers and non-carriers within the alcohol condition at this alcohol dose (*p* = .10) such that, as expected, carriers of the 7-repeat reported increased perceived social bonding compared to 7-absent individuals. Further research is indicated that varies alcohol dose, as higher doses might generate more pronounced effects.

It remains unclear whether 7-repeat carriers possess an actual increased ability to bond with others or if they only perceive their ability to be enhanced. Regardless of this distinction, though, it may be that their perception of increased social bonding is what leads to problematic drinking. Future work is indicated, however, that examines whether 7-repeat carriers are rated as being more sociable by their peers under conditions of alcohol. Research also would be useful to further probe the role of dosage-set, as the present data reveal that placebo participants reported lower PGRS scores than did control participants. This seemingly counterintuitive pattern has been observed for cognitive processes where compensatory mechanisms are implicated [83], but it is unclear how this would apply to our social interaction.

Despite notable strengths, the present study did have limitations. Among these was the fact that the alcohol participants did not differ significantly from the control (no alcohol) participants on the PGRS. This may indicate that a higher dose of alcohol might have been more useful to test. In addition, while the group drinking period started at approximately the same time of day for all participants, we did not control for day of the week, which may have influenced participants' responses. Furthermore, while the distribution of group gender compositions was evenly distributed across the six cells of the experiment, we did not control for this variable and the study was not sufficiently powered to examine its influence on the results. Future studies with even larger samples would permit the examination of potentially interesting three-way interactions including gender and group gender composition as variables. It will also be essential for future studies to test whether carriers of the 7-repeat allele choose to drink more alcohol in social contexts as a result of their perception of enhanced social bonding.

The present findings are preliminary and will need to be replicated. While some argue that genotype-dependent interactions should be the primary focus of alcohol research (e.g. [84]), there is also growing skepticism about the utility of examining gene-environment interactions in the context of addiction and psychopathology. This is mainly due to the fact that some initial, positive gene-environment interaction findings failed to replicate in other samples [85], [86] but see [87], [88]. In general, many of the notable replication difficulties in the literature relate to studies of distal behavioral phenotypes (e.g., depression) and molar environmental moderators (e.g., life events), where layers of methodological variation among studies may yield unstable findings [88]. It is worth noting that, in the case of the serotonin transporter promoter polymorphism (5-HTTLPR) literature, gene-environment interactions in experimental studies (e.g., effects of transporter variation on amygdala response to experimentally manipulated exposures to emotional stimuli) have fared well in terms of replication (see [89]). Still, although the present study utilizes an experimental design and builds upon an emerging literature highlighting the importance of social factors in the association of *DRD4* genotype and drinking outcomes [62], [63], replication is crucial.

In contrast to gene-environment studies focusing on naturally occurring variation in putative environmental moderators and down-stream behavioral phenotypes, studies of genetic influences moderated by experimentally manipulated environmental exposures (as is the case in the present study) have at least two advantages. First, these designs allow for observations to be made under controlled and uniform stimulus conditions, and second, these paradigms better permit causal inferences, because the environmental effect is not subject to contamination by gene-environment correlation [90]. The present study examined a gene-environment interaction in the context of an experimentally manipulated environmental factor, which presumably offers a more powerful tool for identifying gene-environment interactions than do population based studies [81], [90]. A large number of participants received alcohol, and drinking patterns were equivalent across groups. Personality traits thought to relate to social bonding also did not vary across groups. Though such studies raise ethical considerations, it potentially would be valuable to extend these findings in individuals who meet criteria for alcohol use disorders. Additional work that examines other polymorphisms in relation to alcohol-induced bonding and studies that investigate alcohol's effects on non-verbal measures of social-emotional responding throughout a drinking interval also would be useful. Nevertheless, together with other recent findings targeting *DRD4,* these results suggest that interventions may benefit from focusing on social reward as an important underlying mechanism for the development of problematic drinking in a subset of young adults. More generally, our findings highlight the potential utility of employing transdisciplinary methods that integrate genetic methodologies, social psychology, and addiction theory to improve theories of alcohol use and abuse.
